# Magic, Witchcraft, and Animal Magnetism
*An History of Magic, Witchcraft, and Animal Magnetism. By J. C. Colquhoun, Esq. 2 vols. London: Longman, 1852.Isis Revalata; An Inquiry into the Origin, Progress, and Present State of Animal Magnetism. By J. C. Colquhoun, Esq. 2 vols. Edinburgh: Maclachlan and Stewart, 1836.Letters on Animal Magnetism. By William Gregory, Professor of Chemistry in the University of Edinburgh.Observations on J. C. Colquhoun's History of Magic, Witchcraft, and Animal Magnetism. By James Braid, M.R.C.S.E. London: Churchill, 1852.


**Published:** 1852-07-01

**Authors:** 


### MAGIC, WITCHCRAFT, AND ANIMAL MAGNETISM.*

THE REVEREND SYDNEY SMITH, in his Lectures on Moral Philosophy, delivered at the Royal Institution, profoundly observed, that "errors to be dangerous must have a great deal of truth mingled with them; it is from this alliance," he adds, "that they ever obtain an extensive circulation; from pure extravagance, and genuine, unmingled falsehood, the world never has, and never can, sustain any mischief."†

So correct is this observation, that we feel assured that no faith, however extravagant, ever took a strong hold upon the public mind without being in some measure founded upon truth. It may be difficult to separate the grain from the chaff—the pure ore from the sullying dross; it may require great critical acumen to discover the exact point at which the wave of truth blends itself almost imperceptibly with the stream of human imagination, as it runs rapidly into exaggeration and fiction; but that such a line of demarcation may be drawn there can be

* An History of Magic, Witchcraft, and Animal Magnetism. By J. C. Colquhoun, Esq. 2 vols. London: Longman, 1852.

Isis Revalata; An Inquiry into the Origin, Progress, and Present State of Animal Magnetism. By J. C. Colquhoun, Esq. 2 vols. Edinburgh: Maclachlan and Stewart, 1836.

Letters on Animal Magnetism. By William Gregory, Professor of Chemistry in the University of Edinburgh.

Observations on J. C. Colquhoun's History of Magic, Witchcraft, and Animal Magnetism. By James Braid, M.R.C.S.E. London: Churchill, 1852.

† Elementary Sketches of Moral Philosophy. Delivered at the Royal Institution, in years 1804, 1805, 1806. By Sidney Smith, M.A. London: Longman, 1850.—p. 5. So also St. Croix observes: "It is with difficulty we can imagine anything full of improbability—a fact of this nature is rarely forged."—*Examen Critique des Historiens d'Alexandre.* Paris: 1804.—p. 9.

no doubt. Under this view, the history of every superstition is in itself an important chapter in the history of man. Astrology, Magic, Sorcery, Witchcraft,—the very names of these exploded pseudo-sciences, as they are called, may now excite the risible faculties of the modern philosopher. He fears not the approach of Comets, or the wild glare of the "Northern Lights;" the "silent beauty of the starry heavens" no longer excites in his mind any apprehension, or feeling that he is himself a Microcosm connected with the changes and sympathies of the surrounding universe. He knows full well that the Egyptian and Greek temples have been overthrown—the Delphic and Cumæan oracles silenced—the Sybilline leaves scattered to the winds, and that the Eleusinian mysteries have themselves become a myth; but in the midst of all his scepticism, he cannot look through the *vista* of the past—he cannot open the pages of history, whether sacred or profane, without, if he have any element of faith within him at all, believing that such things *did* once exist; that they were not merely idle shadows flitting across and obscuring the human mind, but that they were circumstantial and stern realities, which affected the hopes and fears, the moral conduct and civil responsibilities, and all that could possibly appertain to the happiness of man. True it may be that man is naturally a credulous animal. The very consciousness of his restricted and finite capacities seems to suggest within himself a desire to go beyond the apparently prescribed boundaries of human knowledge. He is not satisfied with analyzing the air he breathes, the earth upon which he treads, the light of heaven; he is not contented with observing the universal and undeviating relation which exists between cause and effect, but must have recourse to some clumsy and hazardous hypothesis to link, according to his narrow notions, the antecedent and consequence mechanically together. Nay, the very consciousness of his own existence—the Cartesian principle—the "*Cogito ergo sum*"*—to his apprehension is an insufficient evidence that he really lives, and moves, and has his being. He would fain anatomize his mind, as well as his body; and because he cannot succeed in discovering how the two are connected together, he turns round upon himself, puts a bold face upon the mystery, and denies altogether the existence of his spiritual nature. Thus does our frail modern philosopher ever lean upon the weakness which betrays him: seeking to learn more than has been vouchsafed for him to understand, and keeping his eyes fixed on the very feeble and flickering lamp of physical science, he goes about constantly groping

* "Who am *I?*" says Thomas Carlyle; "What is this *me?* A voice, a motion, an appearance. Some embodied visualised idea in the eternal mind? '*Cogito ergo* sum?' Alas! poor cogitator! this takes us but little way. Sure enough I am, and lately was not. But whence? How? Whereto?"—*Sartor resartus.* 2nd ed. London: 1841. —p. 61.

in the dark, and, like Faustus, under the guidance of Mephistopheles, in the "Brocken," is liable to be misled by every *ignis fatuus* that flits across his path.

But, after all, is there no fixed and abiding reality in nature?—nothing for the good of man, as Wordsworth finely observes, "more solid than the gilded clouds of heaven?"* Is there no Positivism in Philosophy? Assuredly there is! Truths older than the mountains are still extant, which have survived the marble ruins of the temples wherein they were enshrined. But to read these rightly we must divest ourselves of educational prejudices; we must apprehend no war, but advocate an universal toleration of opinion; so that we may come to the task with a thorough and conscious feeling of mental independence. The fetters that would enthral the mind are infinitely worse than those which may inflict restraint upon the body.

"The cause" of Animal Magnetism, if we may so express it, has been so long before the public,—its pretensions have been so frequently discussed, and the phenomena which are said to be developed by its mystic agency, are so well known, that we need not recapitulate them. But this much we think certain: the physiological and psychological mysteries of our nature, the connexion which exists between life and mind, soul and body, is as little understood now as in the days of Plato and Aristotle. "Thus far shalt thou go, and no farther;" but who is to delineate the exact boundary of knowledge attainable by the progress of science? The most exquisite dissections of the brain and nervous system must ever fail to enlighten us, because the most minute anatomy, even in all its microscopical relations, can never determine the function of any organ. An elucidation of its structural adaptation to a given purpose is all we can arrive at; but man, viewed synthetically, unable to comprehend himself, is nevertheless a being partly material and partly immaterial—having a corporeal and a spiritual nature; and here the observation of Coleridge appears to be peculiarly apposite: "All the organs of sense are framed for a corresponding world of sense; and we have it, all the organs of spirit are framed for a corresponding world of spirit, though the latter are not developed in all alike."†

The "Isis Revelata," the "History of Magic, Witchcraft, and Animal Magnetism, by Dr. Colquhoun;"—"Letters on Animal Magnetism, by Professor William Gregory;" Reichenbach's "Physico-Psychological, on the dynamics of Magnetism, Electricity, Light, Heat, Crystalization, and Chemism," by Dr. Ashburner; Dr. Braid's work in answer to Mr. Col-

* Excursion;—Despondency Corrected. Ed. Longman, 1832.—vol. iv.

† Biographia Literaria. A Biographical Sketch of my Life and Opinions. By S. T. Coleridge: 1819.—p. 27.

quhoun on "Magic, Witchcraft, Animal Magnetism, Hypnotism, and Electro-Biology;"—these, and numerous other works on the same subjects, in German, French, and English, lie, as we have stated, before us; but we are disposed to give Mr. Colquhoun's researches the priority of consideration on the present occasion, because he was, clearly, the first *Littérateur* who called the attention of men of science in this country, to the report made by a committee of the Royal Academy of Medicine at Paris favourable to animal magnetism, in 1831; few copies of which were then in circulation; and because a few years afterwards, in 1836, when he published the "Isis Revelata," he advocated with all the erudition he brought to bear upon it, the cause he had espoused with a degree of modesty and a tone of fervid and persuasive eloquence ever becoming a man, who is at the same time a philosopher and a gentleman. These two characters, be it observed, are not always combined. There is, indeed, an outcry abroad, a popular fallacy than which none can be more pernicious, that all men of science who have been fortunate enough to make great discoveries, have invariably been subjected to ill-treatment, made subjects of ridicule and persecution, and doomed to undergo a species of slow martyrdom. Nothing is more untrue! There can be no analogy whatever fairly drawn between the inquisitorial age in which Galileo lived, and any succeeding age in the history of European philosophy; and if we only read fairly and dispassionately the lives of Harvey, Sir Isaac Newton, and Jenner, we shall perceive that these illustrious men, so far from being martyrs, lived to see their own glorious achievements in science fully recognised and established, and secured all the highest honours which the sovereign, the state, and the universities could confer upon them.* It is, however,

* We have often wondered that this cant, founded obviously upon the grossest distortion of facts, should not have been long ago peremptorily refuted. The persecutions conducted against men like Galileo by these iniquitous tribunals, characterise the barbarism of an age which has long since past away. The war was not so much against science as against the chance of discoveries being made which might shake the pillars of those gloomy abodes of darkness to their foundation. Then what do we find when we look into the lives of Harvey, Sir Isaac Newton, Jenner? The anatomical evidence connected with the circulation of the blood, the structure of the heart, the course of the arteries and the veins, the valvular structure of the veins—nay, the movement of the blood, and even the double—the pulmonic and systemic—circulation, had been discovered before the time of Harvey, and when he accomplished his admirable "*Exercitatio de Motio, Cordis et Sanguinis,*" and by concentrating the whole evidence inductively, irresistibly established the truth of what others had already surmised and believed certain objections and physiological difficulties—such as the inadequacy of the power of the heart to propel the blood directly from the arteries into the veins, and the nature of the capillary circulation—Harvey turned round upon his antagonists in a very intolerant and abusive spirit, heaping upon them the most abusive epithets. But what followed; the sole merit of the discovery was awarded to him in his life time; he was made physician to the king, and enjoyed the highest honours to which a professional man could attain. Then again Sir Isaac Newton—what cause for lamentation have we here? True, Huyghens disputed many of now a days the fashion with every empiric who boasts of having discovered some great secret which he professes will benefit mankind, and which at the same time puts money in his own pocket, to exclaim "Galileo was persecuted ; so am I ! Look at the histories of Harvey, Sir Isaac Newton, and Jenner; how were their discoveries treated, and how are mine?" Will it be believed that the stupid public actually hesitated before it would consent to swallow, when first discovered, such valuable remedies as Peruvian bark, antimony, arsenic, cantharides, and prussic acid? We lament that this kind of jargon ever should be tolerated. It is the language only of men who are writhing under disappointment. We insist that it is the duty of every conscientious man, before embracing any new doctrine, to examine into it thoroughly. He is bound to sift every particle of evidence that can be adduced for and against it, before he comes to any conclusion. Hasty inferences are never sound. The world is never wrong in its ultimate judgment: and we often feel surprised that men of science should so constantly betray an irritability of temper unbecoming the real interests and dignity of philosophy, simply because their discoveries—their theories—their peculiar views, sometimes upon subjects which are in their own nature peculiarly abstruse, are not at once accepted by men of their own intellectual status; and we cannot help thinking that this hastiness and intolerance too frequently betrays the latent existence in their breast of that very spirit of persecution from which they claim protection. It is well for the real interests of truth, that philosophers should see the same facts from different points; and that they should, taking different paths, arrive at different conclusions. We may rest assured that this very conflict of opinion stimulates and energizes the reasoning faculties; tends to clear away prejudices that would otherwise obscure the understanding, and is as essential to the healthful activity of the human mind, as the agitation of the waves of the sea is to preserve the purity of its waters.

The truth is, the world is far more given to credulity than to scepticism. "Man is credulous from the cradle to the grave," and there never yet was any hypothesis ever hazarded, however extravagant, his theorems, but he lived also to see his discoveries admitted into all our schools of philosophy—was recognised as the greatest philosopher of the age, and received all the honours which the existing universities and the Royal Society could confer upon him. Finally comes the case of Jenner. His discovery was doubted—very properly so—the *onus probandi* lay with him; and when this he established—then his discovery too was recognised, and he, too, lived to enjoy not only the honour awarded to him, but the advantages of a large parliamentary grant. The world, we repeat, is more inclined to be over credulous than over sceptical, and we never could understand why sensible persons should be accused of being incredulous because they hesitate before they swallow every new-fangled medicament which ingenuity may suggest.

and *nullum est tam absurdum quod dictum non credat aliquis philosophorum*, that has not speedily found a host of specious advocates suddenly start up in its defence. Nay, men of profound learning and of the highest sagacity have too often allowed themselves to be drawn into the vortex, and carried away by the stream of popular enthusiasm. The professors of animal magnetism have, we conceive, nothing to complain of; they must expect that scientific and literary men will receive the extraordinary statements they submit to them with hesitation and caution. What said Treviranus, the famous botanist, to Coleridge? "I have seen what I am certain I would not have believed on your telling; and in all reason, therefore, I can neither expect nor wish that you should believe on mine."* Seeing, then, that many of the higher phenomena of animal magnetism (as they are called) come before us *primâ facie* with an aspect of improbability, how impolitic it is in the believers of animal magnetism who seek to make converts, to set out with blazoning before the public facts which, if true, appear to be contrary to the experience of mankind and the *dicta* of common sense. Who can believe that a lady sending a lock of her hair from London to a magnetizer in Berlin will thereby put herself *en rapport* with him, and enable him to magnetize her at this distance—nay, to prescribe successfully for any complaint with which she may be affected? Who can believe a story we met with in the "Zoist," that a magnetizer, by breathing into a pair of gloves, and thinking at the same time of some particular subject, may communicate a dream to the person, when asleep, to whom the gloves belonged? We repeat, that it is unwise in men who invite us to believe in their doctrine to set out with facts which, as Treviranus implies, must appear so palpably impossible; for, as Locke truly observed, "There are so many things to be known, and our time on earth is so short, that we must at once reject all useless investigations." But what investigations are useless? or what learning? Upon the subject of animal magnetism, we have before us such a mass of contradictory evidence—so many conflicting opinions—such a tangled and complicated web, that we are not disposed to pronounce judgment; at all events, to borrow the language of our law courts, "without appeal." The facts adduced, however, by Mr. Colquhoun, showing the analogy, indeed, close similarity, between the effects which were produced in past ages by magic and witchcraft, and those which are now produced by animal magnetism, are exceedingly curious. We must, however, adhere to facts alone—*res non verba*. The theories suggested in explanation of them are another matter, and come clearly within another range of science. The physiological and psychological effects produced by the

* Coleridge's Table Talk.—Vol. i. p. 107. Isis Revelata.—Vol. i. p. 76.

Egyptian magi and Greek priests on their willing and credulous votaries, have been circumstantially described by historians, who could have had no conceivable or possible motive to distort or misrepresent facts which were of every-day occurrence, and patent to the observation of the multitude. We have only to refer to the records of Biblical history to convince ourselves that the magicians were held in the highest estimation; that they were consulted in all cases of emergency by the most exalted, learned, and highly-gifted—we might almost say, heaven-inspired—men of the age; and that these "wise men," as they were called, possessed the art of performing many apparent miracles. Their first appearance, as recorded in the Holy Scriptures, was on the occasion of Pharaoh's two dreams of the seven years of plenty and seven years of famine. We read (Genesis xli. 7, 8), "And Pharaoh awoke, and behold it was a dream. And it came to pass in the morning that his spirit was troubled, and he sent and called for all the magicians of Egypt and all the wise men." Their second appearance was on the memorable occasion when Moses and Aaron, armed with miraculous power, cast the rod before Pharaoh, which became a serpent. "Then Pharaoh also called the wise men and the sorcerers; now the magicians of Egypt they also did in like manner with their enchantments," (Exodus vii. 11.) Their power of enchantment, divination, and interpreting dreams, is frequently attested. Twice did Nebuchadnezzar, King of Babylon, "command the magicians and the sorcerers and the Chaldeans for to show the King his dreams;" and because Daniel interpreted his dream, "the King made Daniel a great man," and the "chief of the governors over all the wise men of Babylon," (Daniel ii. 2—49.) The art of divination may be traced to the same early period, for in the history of Joseph, in the Bible, we read that after Joseph had entertained his brethren, who "rewarded evil for good," he sent his steward after them to bring back his silver cup, desiring him to accost them in these remarkable words: "Is not this it in which my lord drinketh, and whereby he divineth?" (Genesis xliv. 5.) From this story, "it plainly appears," as Godwin observes, "that the art of divination was extensively exercised in Egypt, that the practice was held in honour, and that such was the state of the country that it was to be presumed as a thing of course that a man of the high rank and distinction of Joseph should be an adept in it."* To return to the magicians. "They were," says Mr. Colquhoun, "in these early times held in the highest estimation by mankind, as the venerated depositaries of all science, sacred and profane—consequently as the mediators between earth and heaven, the interpreters of the divine will to the

* Lives of the Necromancers. By William Godwin. London: Mason, 1834. p. 48.

inhabitants of this lower world. Their social rank corresponded with the dignity of their sacred functions. They were either themselves princes of the land, or the chief tutors and indispensable councillors of princes, as we learn from the Old Testament and other records. As their duties, however, were paramount, so were their responsibilities great and stringent. The qualifications required from them, in addition to learning and practical wisdom, were strict devotion to truth and justice, and a pure disinterestedness of moral character. . . . . Among the Persians, the magicians, as in other countries, presided over the sacerdotal office, and magic thus became combined with religion. Plato, in his 'Alcibiades,' informs us that the kings of Persia learn magic, which is a worship of the gods. Magic, therefore, in those ancient times, had reference to everything which was supposed to relate to human and divine science—to medicine and to philosophy, as well as to religious worship."* At an early period the office of priest was always combined with that of physician: the Hebrew priests were physicians among the Jews; the Asclepiadæ, or priests of Æsculapius, were the first physicians of the Greeks; and the Druids those of the northern nations. So also among the Naodëssis and Chippeways, the three titles of priest, physician, and sorcerer, were inseparable, and they are still so among the Osages. In Mexico the priest-magicians were for many years the only physicians. Hence medicine was originally cultivated as a branch of occult science, and practised in the temples wherein magical rites and magical formularies were had recourse to. It was from Egypt that the formularies which taught the use of herbs were derived, as well as many medical substances: but these formularies and substances were magically applied. The magicians of the Island of Sena cured persons who were by others deemed incurable. The Scandinavian virgins were at the same time instructed in magic, medicine, and the treatment of wounds. We ought also here to remark, as we shall have occasion to revert to the subject, that astrology, which was coeval with astronomy, also came within the range of magic, which, in fact, embraced all sciences. Accordingly we are not surprised to find the most illustrious philosophers travelled into India and Egypt in pursuit of this knowledge, and devoted themselves to its study. Cornelius Agrippa, in his "Occult Philosophy," observes, that "Pythagoras, Empedocles, Democritus, Plato, and many other renowned philosophers, travelled far by sea to learn this art, and, being returned, published it with wonderful devoutness, esteeming of it as a great secret. Also it is well known that Pythagoras and Plato went to the prophets of Memphis to learn it, and travelled through almost all Syria, Egypt, and Judæa, and the schools

* Hist: vol. i. p. 11—127.

of the Chaldeans, that they might not be ignorant of the most sacred memorials and records of magick, as also that they might be furnished with divine things. Whosoever, therefore, is desirous to study in this faculty, if he be not skilled in natural philosophy, wherein is discovered the qualities of things, and in which are found the occult properties of every being; and if he be not skilful in the mathematics, and the aspects and figures of the stars, upon which depends the sublime virtue and property of everything; and if he be not learned in theology, wherein are manifested those immaterial substances which dispense and administer all things—he cannot be possibly able to understand the rationality of magick. For there is no work that is done by meer magick, nor any work that is meerly magical that doth not comprehend these three faculties."*

The secrets of magic, and the mystical rites and ceremonies practised by the Chaldean and Egyptian magicians, were thus communicated and carried into the Greek and Roman temples; and this brings us into the very heart of these mysteries—the solemn oracles delivered by the Pythian priestess, whose voice was listened to with awe and fear—the inspired Sibyls, whose prophetic volumes, when lost, the Roman augurs and the Roman people themselves would have given worlds to recover.† Then come the phenomena developed during the temple sleep, which fell upon all those who went to consult the oracle, and who passed into a state of what is considered ecstatic sonnambulism—all which are very curious subjects for investigation. But to proceed; when Chris-

* Three books of Occult Philosophy, written by Henry Cornelius Agrippa, of the Nettesheim, Counsellor to Charles the Fifth, Emperor of Germany, and Judge of the Prerogative Court. Translated from the Latin into the English tongue, by J. F. London: Printed for Gregory Moule, and are to be sold near the West end of St. Pauls, 1651.—p. 5.

† "Great was the veneration conceded to the sibyls in Greece and Rome; in proof of which we need only cite the Sibylline volume, to discredit which, in the olden time, would have been a matter of danger. It is known to all that a venerable sibyl came to Tarquin and offered to sell him nine volumes of her prophecies. When her price being taxed as exorbitant she threw three of the volumes into the fire, still requiring the same price for the remaining six. Still denied her price by Tarquin, three more of her books shared the same fate; and on her adhering to her original demand for the remaining three, Tarquin assembled the augurs, who advised the purchase, and the monarch was obliged to submit to the terms of the sibyl. From that moment the Sibylline leaves became objects of veneration. They were handed over into the custody of the priests, and consulted upon occasions of importance after a decree of the senate. These volumes were destroyed in the conflagration of the Capitol, eighty-three years before Christ—a severe calamity to the Romans, who looked upon the Sibylline books as a sacred charta. It is remarkable that after the destruction of these volumes the republic gradually declined and fell under the yoke of the emperors. Immense as was the loss of the volumes, considering their influence over the minds of the people, the augurs and senate hoped to replace the loss. Zealous missionaries were sent to all the cities of Europe, Asia, and Africa, which affected to possess sibylline verses, and more than two thousand were brought back. But we are to conclude they were far from genuine, as under their consultation the sibylline oracles declined in credit. Augustus suppressed tianity became fairly established, the temples of the heathen* were overthrown, or converted into Christian churches;† their altars, which had been profaned by pagan rites, shattered, or consecrated and appropriated to the service of a holier and purer religion. Then the aspect of the civilized world underwent such a change as we behold when the many of the verses, and the rest were burned by Stilicon, father-in-law of the emperor Honorius." (World of Wonders, pp. 120, 121. This book contains an interesting account of a variety of ancient and modern superstitions.) Mr. Colquhoun, who appears to have consulted an immense number of classical authorities on the subject, tells us that "little is known with certainty in regard to these sibyls. Even their exact number and names have been subjects of controversy," and "their precise number cannot now be determined." . . . . "The sibylline books were consulted in cases of disease as well as in important affairs of state, and were particularly respected at Rome as the tutelary guardians of the empire."—Hist. v. i., p. 200.

* We do not, be it observed, use the word "*heathen*" in a derisive sense. To us it appears that the pagan religion, and all the multifarious creeds and forms of worship adopted in the East, were permitted by the Supreme Being to prevail for good and wise purposes, albeit we may not be able to fathom them. On this subject Mr. Colquhoun observes (and we think every conscientious Christian must agree with him), "The pagan religion, it is true, was full of superstitions, and every sane man must admit that the Christian scheme is in every respect infinitely preferable to that which it superseded. . . . . But what rational being can believe that the Creator could have abandoned his creation to itself during four thousand years? Is it not more natural to conclude that if the Pagan religion was not more distinguished by its simplicity and its purity, it was because the Deity, in his infinite wisdom and mercy was pleased to wait until mankind, by contemplation and reflection, had time to elevate themselves to a purer faith, and, like the Hebrew nation, should come to adore him everywhere in the universe, without confining him to any particular spot." (Hist. vol. i. p. 215.) Further on he remarks, "The religion of the pagan world no doubt was full of the grossest and most degrading superstitions, and was utterly incapable of satisfying the minds or awakening the consciences of the more elevated classes of the people among whom it prevailed. Nevertheless, it was infinitely preferable to a total want of all religious faith and worship, and an indifference to those moral counsels and injunctions which are believed to emanate from a superior world. Nor ought it to be forgotten that this lower sphere of existence required to be prepared in some measure for the advent of Jesus Christ." (Hist. vol. ii. p. 2.) Many of the fathers of the church took this view. For our own part, we never could perceive the arch heresy of those lines in "Childe Harold," which an over fastidious critic in a quarterly review so vehemently reprobated:

"E'en gods must change—religions take their turn;
'Tis Jove's—'tis Mahomet's; and other creeds
Will rise with other years, till man shall learn
Vainly his incense soars, his victim bleeds."

The fact is an historical one; and even Pope, in "The Universal Prayer," which has always been admired for its fervent piety, expresses, but not so clearly, the same view—

"Father of all, in every age,
In every clime adored
By saint, by savage, and by sage—
Jehovah, Jove, our Lord."

There are, however, some hypercritics who maintain that Pope's "Essay on Man," "The Universal Prayer," &c., are only versifications of what they are pleased to call Lord Bolingbroke's "shallow and hollow sophistry."

† *Vide* "Vestiges of Ancient Manners and Customs, discoverable in Italy and Sicily." By the Reverend John James Blunt, Fellow of St. John's College, Cambridge. London: 1832.

morning sun dissipates the mists which sometimes obscure the fairest prospects upon earth. But it is contended that the same natural causes still remained in operation, and that during the middle ages the arts of sorcery and witchcraft re-developed the same effects which formerly had been exhibited in the ancient temples. Finally, the belief in sorcery and witchcraft has been generally exploded throughout Europe; and now animal magnetism appears upon the stage, and certainly it does not come before us as these heathen rites and ceremonies did—the visible symbols of the most extravagant superstitions; nor is it intruded upon us as sorcery and witchcraft were by the vulgar, under a cloud of impenetrable ignorance: but it assumes a more seductive aspect, pointing to curious and anomalous facts which have been observed by medical men in all ages, and inviting us to view these fairly in a psychological light.

"One of the great advantages," says Mr. Colquhoun, in the "Isis Revelata,"* "attending the study of animal magnetism, is, that it tends to approximate the sciences of physiology and psychology—the phenomena of the material with those of the spiritual man—by demonstrating experimentally the intimate connexion that subsists between them. The study of physiology has of late been generally confined to an investigation of the component parts and mere material structure of the organism, with little or no regard to the principles which regulate their action in living beings. Psychological science, strictly so called, on the other hand, has been for a long time greatly neglected in this country, and its phenomena, even when they presented themselves to notice, have been almost entirely disregarded, although of paramount interest to every intelligent living being, and of the utmost importance to the philosophy of man." This, be it observed, was written six years ago; since which period we have had the satisfaction of observing that the study of psychology, particularly in connexion with insanity and cerebral pathology, has become general. We cannot expect, as in the physical sciences, to make discoveries which shall be at once demonstrable and startling to the public mind. The analysis of the mental faculties cannot be exhibited, as Messrs. Brande and Faraday may exhibit the wonders of electro-magnetism at the Royal Institution. Here are no coruscations of light to be developed—no brilliant phenomena, which, by striking the eye, may arrest and entrance the attention; nevertheless, psychological science is progressing, and if animal magnetism could only assist in unveiling to us—as its sanguine professors promise—the laws which govern our spiritual nature, assuredly it

* Introduction. Vol. i. p. 24.

would be universally cultivated; for speculations of this description have been ever uppermost in the mind of man. But here is the *questio vexata*, the great stumbling-block, intercepting us on the very threshold of its temple.

The object of Mr. Colquhoun's "History of Magic, Witchcraft, and Animal Magnetism," is to prove that animal magnetism will explain all those miracles and mysteries which have been recorded in history: the magicians and heathen priests produced all the wonders ascribed to them, he assumes, by virtue of animal magnetism; sometimes by silent volition, sometimes even by manipulation; they were all, in reality, animal magnetizers—Moses, Aaron, and the prophets. "The prophetic views of Moses," he tells us, "were either the result of magical or magnetic intuition, in consequence of a natural predisposition to the ecstatic affection, an idiosyncracy which appears to have been characteristically prevalent among the Jewish nation; or they were the effects of the immediate influence and inspiration of the Almighty; or both causes may have been combined." "The Scriptures abound with allusions to the magnetic treatment and phenomena, the prophetic dreams and visions of the Jewish patriarchs and seers were manifold."* "The miracles of the magicians, the Delphic and other oracles, the prophetic inscriptions of the sibyls, the temple sleep, the cures wrought by the Asclepiadæ—these were the triumphs of animal magnetism in those ages." But let us pause. It is only fair to observe, that other believers in animal magnetism may not esteem those views canonical; they are not essentially a part of the mesmeric code; and many who have faith in its doctrines may repudiate this application of them. We are not, be it observed, discussing the truth or falsehood of animal magnetism, but the explanation to be given of the historical evidence before us. And here we would urge upon the reader to keep steadily in view the susceptibility of the mind to be affected by impressions from without; and when these are of a solemn and imposing character, they may excite emotions which will act directly upon the whole physical organism, and give rise to effects which it is impossible to predicate. Abnormal effects may be produced when the spiritual attains ascendancy and power over the physical nature of man, which are in themselves exceedingly extraordinary. And it appears to us, that this direct action and influence of the mind upon the body will sufficiently account for such results, without having recourse to any magnetic fluid, or odyle force, or any other interposing material element, however exquisitely attenuated it may be supposed. The miracles performed by the magicians, and many recorded in the Old

* History, vol. i. p. 1.

Testament, cannot, we conceive, be brought in any way to dovetail with the magnetic theory. We cannot conceive how any amount of magnetic fluid—not even Reichenbach's "od force," with all its blue lights, could have converted the rod of Moses into a serpent—turned the river into blood; or how any magnetic influence they might have possessed would have enabled the magicians (Exodus viii. 7,) "with their enchantments, to bring up frogs upon the land of Egypt." The great contention for supernatural power between Moses and the Egyptians was designed obviously to show the people of Israel that the Almighty invested Moses with powers beyond those which the magicians could command. And these miracles doubtless were intended to vindicate the great power of the Almighty, and convert the Israelites who witnessed them, from their idolatry. We cannot perceive what the assumed "magical or magnetic intuition" of Moses could have to do with summoning up reptiles, and casting other plagues on the land of Egypt; nor do we admit that both causes may be combined. We next come to the magnetic theory of the interpretation of dreams. Here, again, we cannot perceive how the principles of animal magnetism could have been applied. When the magicians were called upon to interpret the dream of Pharaoh—when Daniel was commanded to interpret the dream of Nebuchadnezzar, the dream, be it observed, in each case, was at an end. The dream of Pharaoh had passed, and "in the morning his spirit was troubled." The dream of Nebuchadnezzar was forgotten, and his "spirit was troubled to know the dream." The magicians, astrologers, sorcerers, and Chaldeans were thereupon sent for, to show the king his dreams, and interpret them, which they failed in doing, but which Daniel accomplished. How can the doctrine of animal magnetism, we ask, be in either of these instances applied? If the dream-interpreters in either case had been introduced into the presence of the sleeper, and described what was passing through the dreamer's mind, and the signification of the same, the examples would be more apposite; but when these oneiro-critics were called in to propound its meaning, the dream had flitted past—the relation between the interpreter and the dreamer—the magnetizer and subject of magnetization, had not, upon magnetic principles, been established; there could have been no *rapport* between them; no thought-reading of thoughts that had passed away and were forgotten. The oneiro-criticism, or interpretation of dreams, was always a branch of occult science, but animal magnetizers do not profess to possess this faculty, and do not, we presume, go about expounding dreams.

The connexion between animal magnetism and the oracles of antiquity next claims our notice. These oracles were very numerous.

The chief was that of Delphi; there were, besides, oracles of Zeus, Apollo, and other gods, and many were erected to Æsculapius, the most celebrated of which was the temple at Epidaurus. This, Mr. Colquhoun has specially selected, in order to exhibit the particular procedures adopted in these temples. We must, our space being limited, abbreviate his very graphic description, and request the reader to consider, as he proceeds, the effects, independent of animal magnetism, likely to be produced upon any mind, even the best informed in the present day, by such scenes and ceremonies as are here described. "Epidaurus is said to have been the birthplace of Æsculapius; and for this reason it was held to be peculiarly sacred. Multitudes of patients flocked to this temple, in order to recover their lost health, and to become enlightened by divine dreams. The temple itself was situated in a beautiful spot upon a considerable eminence. On all sides it was surrounded by wooded hills, where the air was exceedingly pure, and there was abundance of excellent spring water. The charms of nature were enhanced by beautiful artificial groves and pleasure walks, and even enchanting spectacles. Behind the temple stood the dormitory for the patients, and near it a round marble bath. In the temple itself there were many antechambers, and in the very innermost recess the statue of the god. . . . . No person, unless on very rare and uncommon occasions, was admitted into the interior of the sanctuary; the priests alone had access to the presence of the deity. Sometimes strangers were not even permitted to approach the temple. Those who desired access to it, were obliged first to prepare themselves in the neighbouring temple of Isis. In the antechambers of the temple were many votive tablets containing descriptions of diseases and the remedies successfully exhibited. These were sometimes engraved on the pillars of the temples. . . . . Upon entering the Temples the patients were bound by the most solemn promise to pay implicit obedience to the orders and prescriptions of the superintending priests. Abstinence in regard to diet was strictly enforced, and especially from the use of wine. The priests conducted the patients through the antechambers of the temple, pointed out to them the images and votive tablets, and related the miraculous cures which had been performed through the aid of the presiding tutelary deity. Prayers were offered up and sacred hymns were sung, the latter frequently accompanied by instrumental music; and sacrifices were made for the purpose of conciliating the favour of the patron god. Baths were always employed as a part of the preparatory treatment; as also the drinking of pure water. The baths were usually accompanied with frictions, and with various manipulations and anointments (the magnetic treatment). These frictions and manipulations were cautiously administered by individuals specially appointed and trained up and indoctrinated for that particular purpose. Fumigations were also employed previously to admission to the oracle. The object of all the preparatory ceremonies and observances generally, was to induce sleep; and when this disposition was manifested, the patients were laid to sleep, frequently upon the skin of a new-slaughtered sheep (*incubatio*), in the usual dormitory. This temple-sleep or incubation, however, according to Pausanias, generally took place at night, in the different apartments of the dormitory, in darkness and solemn silence. . . . . In this temple-sleep, as in the mesmeric crisis, dreams and visions occurred, and the prophetic faculty was developed in a manner similar to that which is occasionally elicited by the magnetic treatment. . . . . Some of these temple-sleepers not only prophesied, but composed and recited very beautiful verses, a talent which, as we formerly observed, has been occasionally exhibited by the insane, as well as by somnambulists and ecstatics."*

The inferences which Mr. Colquhoun deduces from this and similar accounts, which are clearly enough authentic, are, that the patients who went to consult the oracles on the subject of their health, slept during the night in the temple of Æsculapius, where, during the darkness and solemnity of the surrounding scene, they were magnetized by the priests. He adds, "It is now well known that a particular place, a particular apartment, may be specially magnetized, and somnambulism thus rendered infectious;" and that in the ancient temples there was a particular place appointed, a special apartment, a dormitory, where the patients slept, and under "these circumstances were manifested all those curious phenomena which have astonished, puzzled, bewildered, and perplexed philosophers in all subsequent ages."

Before commenting on the inferences which Mr. Colquhoun has here drawn, let us cross over to the southern side of Parnassus, and enter the chief temple of Apollo—that of Delphi, which was the most celebrated of all the Greek oracles. This temple, too, which was said to have been formed originally of laurel branches only, but which was afterwards converted into a more solid and lasting edifice of stone, was also beautifully situated. The internal arrangements were very similar to those in the temple of Æsculapius; different apartments being provided for the sick, and for those who merely came to consult the oracle. "The Pythia herself had a distinct and separate

* History, vol. i. p. 172, *et seq.*

apartment, into which no person whatever was admitted; and, near to this, was a small cabinet, where those who came to consult her awaited her responses. The open entrance to the cell appropriated to the Pythia was entirely covered with laurel leaves, so that no one who approached it could perceive the prophetess. Among plants, the laurel, as is well known, was particularly sacred to Apollo; and it was believed to possess the property of inducing sleep and dreams.* In early times, young women were for the most part selected for the prophetic office; the Pythiæ (from the word Pythius, a soothsayer) were young and often beautiful girls, of simple manners, chosen out of the lower classes of society. The apartment of the oracle was situated over a chasm, from which issued intoxicating vapours, and she sat upon a *tripos,* or three-legged stool, perforated with holes, immediately over the aperture through which these vapours rose. The Pythiæ were frequently obliged to be changed, on account of the deleterious influence of the gas on their constitutions; indeed, some of them fell victims to its deleterious influence, although they prepared themselves, before ascending the *tripos,* by fasting three days, and bathing in the Castalian fountain. Plutarch informs us that the Pythia, in her delirium, has leaped from the *tripos,* been thrown into convulsions, and after a few days has died.† Having mounted the tripos, the intoxicating vapours soon began to take effect; her figure seemed to enlarge, her heart panted, her bosom swelled, her voice grew more than human, and in this state of ecstatic delirium she uttered wild and incoherent phrases, which were supposed to flow from inspiration. In her appearance supernatural,‡ she was listened to with devout awe; and every word she uttered was supposed to convey a prophetic meaning. We care not to examine how far these predictions were verified; it is sufficient to know they were universally accredited; the question suggested by Mr. Colquhoun's history, is, whether the effects which really were

* History, vol. i. p. 179.

† Plutarch. Orat. Def., c. 51.

‡ The classical reader will remember Virgil's description of the Pythoness, "Deus, ecce Deus," thus paraphrased by Dryden:

"He comes—he comes—behold the God! While thus she said,
(And shivering at the entry staid,)
Her colour changed—her face was not the same;
And hollow groans from her deep spirit came;
Her hair stood up; convulsing rage possessed
Her trembling limbs, and heaved her labouring breast.
Greater than human kind she seemed to look;
And with an accent more than mortal spoke.
Her starting eyes with sparkling fury roll;
And all the god came rushing on her soul!"

Æneid, lib. vi., v. 47.

produced, are to be accounted for subjectively, or by animal magnetism?—that is to say, whether the combined action of a variety of external causes so affected the mind as to cause it to pass subjectively into various abnormal conditions, which consentaneously re-acted upon the body?—or, whether a subtle magnetic fluid was put into operation, which, penetrating the organic system, affected the senses, causing the mind to enter into new and more lucid relations with the surrounding world? All the objective or extraneous circumstances must be fairly taken into consideration. We must suppose that the interior of the temple presented a most imposing and solemn aspect;—and who ever entered any Gothic cathedral, or any great ruin, like the Colosseum at Rome, and looked around him, without being deeply impressed with a sense of awe and of devotion?* We must remember that the suppliant seeking the aid of the oracle, whether in health or in sickness, was so far already victimized by his own faith, that he believed firmly in the supernatural powers of the magician or priest, and had only to close his eyelids to behold the vision he was told would infal-

We confess we prefer Wordsworth's description of Laodamia even to Virgil's:

> "So speaking, and by fervent love endowed
> *With faith, the suppliant heavenward lifts her hands;*
> *While, like the sun emerging from a cloud,*
> *Her countenance brightens, and her eye expands;*
> *Her bosom heaves and spreads, her stature grows,*
> *And she expects the issue in repose.*
> *O terror! what hath she perceived? O joy!*
> *What doth she behold?"* * * *

And how finely does this picture contrast with that of Hermes!

> "Peace, he said.
> She looked upon him and was calmed and cheered.
> The ghastly colour from his lips had fled;
> In his deportment, shape, and mien appeared
> *Elysian beauty, melancholy grace,*
> *Brought from a pensive though a happy place.*
> *He spake of love, such love as spirits feel."*
> * * * * *

There are few finer poems in the English language than the "Laodamia," by Wordsworth.

* Milton, in the well-known description of "the high embowed roof," "antic pillars," and painted windows of a cathedral, casting around a "dim, religious light," had clearly in view the ecstatic visions which a state of high religious fervour might excite:

> "Then let the pealing organ blow
> To the full-voiced choir below,
> In service high and anthems clear,
> As may with sweetness through mine ear
> *Dissolve me into ecstasies,*
> *And bring all heaven before my eyes."*

Young, in his "Night Thoughts," alludes to the exaltation of the mind during prayer, and describes the suppliant as

> "Man in audience with the Deity."

libly appear. We must well consider the effects of the preparatory ceremonies—the absolute solitude, the profound tranquillity, the prolonged abstinence, the bathing, accompanied by frictions and anointment with unguents, which were composed of very active ingredients, while perfumes and fumigations, intended to affect the senses, were diffused through the atmosphere; we have only to bear these preliminary proceedings in mind, and recollect in addition that at the same time prayers were being offered up, sacred hymns sung, accompanied with exquisite instrumental music, while sacrifices were being made to propitiate the favour of the patron god, and we must admit the existence of every predisposing cause to produce sleep, dreams, and visions. But this is not all; the magicians and priests were adepts in every branch of practical science. They could, by the aid of plane and concave mirrors, which were made of steel, silver, or a composition of copper and tin, produce a variety of optical illusions; they could terrify the beholder, by calling up the apparition either of gods or men; they were well acquainted with the principles of acoustics, and could at pleasure not only make thunder reverberate from the roof round the walls of the temple, but constructed ingenious instruments the sounds of which resembled the human voice. They were also expert ventriloquists, and, without being observed to move their own lips, could make sounds appear to issue from the altar, and their statues speak in the name of the gods they represented.* Add to all this, that although the mechanical knowledge of the ancients was not extensive, they carried the art of mechanism to very great perfection; and many of the wonders performed in the temples were achieved by mechanical agency. Their self-moving automata, indeed, were constructed with singular ingenuity; witness the wooden dove, so wonderfully constructed by the philosopher Archytas, that it flew, and sustained

* The learned Eusebe Salverte has, in his work on the Occult Sciences, proved that the ancients were well acquainted with acoustics, optics, hydrostatics, and with many chemical substances, as pyrophorus, phosphorus, naphtha, and alcoholic liquids. They used compositions similar to gunpowder, whereby they produced thunder and lightning; and had recourse to a variety of expedients whereby they on a sudden produced the development of light, heat, and flame. The marvellous philtre—the blood of Nessus—which Dejanira poured on the tunic of her inconstant husband; the poison poured by Medea upon the robe which she sent to her rival, he presumes was a phosphuret of sulphur; thus he ingeniously enough explains how all these apparent miracles were performed. The wand of science dispels the whole mystery. But in some instances we are inclined to think that Salverte, in endeavouring to account for everything, strains his hypothesis; at all events, he passes over in silence the physiological and psychological effects which were unquestionably developed, which are not the less interesting because they were artificially produced.—See the "Philosophy of Magic Prodigies and Apparent Miracles;" French; and the English translation, with notes, by Anthony Todd Thomson, M.D. 2 vols. London, 1846. Also Sir David Brewster's "Letters on Natural Magic." Family Library, No. xxxiii.

itself for some time in the air; also their moving floors. It is even supposed, from examining the pavement in the ruins of the temple of Ceres at Eleusis, that the floor of this sanctuary was double, the upper having grooves and pulleys whereby it might be raised up and down, to and fro ; while underneath the latter was a vault, destined to admit of the action of the necessary machinery. Finally, they were acquainted with many secrets in chemistry; they could make and give different colouring to artificial fire; they could change fluids visibly from one colour to another—from azure blue to purple, from purple to deep red; and could, when they wished to announce an impending calamity, make the vases on the altar of the temple appear to overflow with blood.

The mystic rites enjoined by the pagan religion were in themselves, doubtless, sufficiently imposing; but when, in addition to these, we find the magicians and priests availed themselves of the resources of practical science with which they were familiar, in order to fill their temples with aural and visual illusions, we cannot wonder at the results produced—that the senses should be deluded, and the mind thereby deeply and strangely affected. We must, indeed, take into consideration all the exciting causes. The object, or at any rate the effect, of the preliminary treatment prescribed in all the temples was, to subdue the physical forces which sustain the health of the body, so that it could no longer resist any powerful mental impression. Every physician knows that a healthy and vigorous state of the constitution will enable it to ward off many morbid feelings and delusions which sick fancies will engender; but here we find the temple-sleepers preparing themselves for their dreams and visions by a preliminary discipline, which could not do otherwise than prostrate the energies of the nervous system. Look at the Pythoness. We all know that certain narcotics and intoxicating beverages act as powerful neuro-stimulants: opium, haschish, stramonium, aconite, the juice of the sun or lotus plant (the Soma drink), evidently have and will still produce extraordinary dreams and visions. But here we must picture to ourselves a young officiating priestess, after undergoing the mystic and secret rites of self-preparation, conducted with much ceremony to the sacred *tripos*. She mounts it with great solemnity, and, while inhaling vapours far more intoxicating than the nitrous oxide gas, becomes delirious:* her efforts are preternatural, her gestures vehement, her voice ever and anon fails her,

* The vapour which issued from the mouth of the cavern was supposed to be carbonic acid gas; but Dr. A. T. Thomson observes, that gas "is not sufficiently intoxicating, and I suspect the gas was sulphurous acid, as it caused almost frantic delirium. The secondary effects experienced by the Delphic priestesses were vertigo, nausea, and great weakness of the lower extremities."—*Note in Salverte*, vol. i. p. 165.

and, amidst sudden breaks and abrupt pauses in articulation, she pours forth her half-articulate prophecies; and sometimes, completely overpowered, falls senseless to the ground. "Inebriated with the gas (says Salverte) that exuded beneath the *tripos*, the Delphic priestess fell into a nervous, convulsive, and ecstatic state, against which she might struggle without being able to regain her self-possession. Whilst out of her senses, and under the sway of an over-excited imagination, she uttered some words, or mysterious phrases, from which it was the care of the priests, who stood around her carefully recording every syllable, to extract the revelations of the future. All this is as natural as the sinking languor which succeeded this excessive disorder of body and mind, and which, sooner or later, proved mortal."*

The mental phenomena which are unquestionably developed in a state of high ecstatic mania, which Hecker shows characterized, more or less, all the mental epidemics of the middle ages, and which in our own times have been witnessed in the religious enthusiasts who attended the ministrations of Wesley and Whitfield, are in the highest degree interesting to the psychologist and the physician. This state of ecstasy, the existence of which cannot be doubted, Mr. Colquhoun considers to be identical with analogous states produced by animal magnetism; and here therefore we come to the *questio vexata*—are such mental phenomena to be accounted for upon psychological principles, or are they to be ascribed to the existence of this very subtle magnetic fluid? When we review all the evidence, we cannot help coming to the conclusion that the impressions made upon the mind by the rites and ceremonies described were quite adequate to excite a succession of subjective impulses, which would cause the mind to pass through all these abnormal phases. We observe the same in insanity;—the mind, energizing within itself, in accordance with its own active principles, becomes highly excited. Who has not heard the poor lunatic pouring forth his prophetic rhapsodies: his conceptions are more vivid, his imagination more active, than during his lucid intervals. But what light can the existence of any imaginary material fluid throw upon the mental pathology of his disease? We can conceive the mind subjectively stimulated into such conditions; but the influence of no fluid, however ethereal, can explain to us the phenomena of thought. The vital fluid, the nervous fluid, the magnetic fluid, are supposed to be all modifications of each other, just as light, heat, electricity, and mineral magnetism; and Mr. Colquhoun, availing himself of the interesting researches of Reil, Autenreith, Humboldt, Burdach, Bichat, and others,—demonstrating, not only the secretion and circulation of a

* Salverte, *op. cit.*, vol. i. p. 165.

nervous fluid, but suggesting the probability that this fluid is capable of an external expansion, which takes place with such energy as to form an atmosphere, or sphere of activity, similar to that of electrical bodies,—argues that it is not straining the hypothesis too far to presume that it is capable of being transmitted or directed onwards, either involuntarily or by the volition of one individual, with such energy as to produce certain real and perceptible effects upon the organism of another, in a manner analogous to what is known to occur in the case of the torpedo, the gymnotus electricus, &c.* Since the publication of the "Isis Revelata," the Baron Reichenbach is said to have discovered a principle more subtle than the magnetic fluid, which he has designated the "od" force, or the odyle. This new imponderable was observed by some of his very sensitive and nervous patients, whose acuteness of vision was such that they observed luminous emanations, like small flames—white, yellow, blue, red, and green—proceeding from the poles of the magnet. These researches have been followed up by Dr. Gregory and Mr. Lewis; and it is now affirmed that this luminous fluid—which escapes the observation of persons not endowed with a very high degree of visual sensibility—during the process of animal magnetism, may be seen emanating from the fingers of the operator. "The degree of augmentation of visual sensibility (says Dr. Gregory) must vary exceedingly. I have met with several persons who could, in their ordinary state, see blue light or grey emanating from the ends of my fingers, when I was in the act of mesmerizing. I could enumerate twenty persons who in their ordinary waking state could see these emanations from my fingers, and some of them from my eyes and my forehead. To my mind (he adds) the fact is sufficiently established, that from the functional extremities of the nerves of living beings a fluid, bearing some analogies to the magnetic fluid, emanates the more abundant as thought and will are modified."† We believe thoroughly in the good faith of Dr. Gregory, and have no doubt that *he* saw, or imagined he saw, these luminous emanations; but they may, nevertheless, have been only optical illusions, for by intensity of gazing an irritation might be excited in the visual organs, and an irregularity in the circulation of blood through the vessels of the retina, which, in all probability, would satisfactorily account for the phenomena. Many persons subjected to head-ache from determination of blood to the brain, shutting their eyes, will see streaks and sparks, and globes of fire; but supposing that the "od"‡ force really did exist, with all its lu-

* Isis Revelata, vol. i. p. 182; vol. ii. p. 151.

† Letters on Animal Magnetism.

‡ The "Zoist" takes up arms against this subtle enemy, and relaxing into a spirit of unusual levity, scarcely becoming a state of mesmeric coma, cites some terminal lines from the stanzas of a legend entitled "*The Lay of St. Odille,*" by the late Thomas minosity, it would only prove the development of a very subtle imponderable fluid in the human body, akin to what is considered to be the nervous or vital fluid. The hypothesis that an ethereal fluid pervaded the universe, establishing inter-stellar relations between distant planets, and diffusing itself, not only through the particles of inorganic, but of organic matter—the pantheistic notion that it was an emanation from, or extension of, the Deity himself, infusing vitality into all created beings, constituting in itself the soul of man, is very ancient. It was the "Anima Mundi" of the Greek philosophers, some of whom maintained

Ingoldsby, Esq. The "Zoist" speculates that the word "*od*" [not spelt as the word *odd*, though the whole thing is *odd* enough,] may have occurred to Baron Reichenbach from the name of the Teutonic god "Odin." But Mr. Thomas Ingoldsby knew more of the veritable origin of saints than most of their biographers. Whence, then, the derivation of the name "Odille?" The "Zoist" says, "there was once a saint of this name." Q.E.D.! "Mr. Barney Maguire (says Ingoldsby) has laid claim to the *next saint* as his countryman; and why wouldn't he? When all the world knows the Odells [not presuming to have descended from Odin] were a fine ould ancient family—sated in Tipperary,

'Ere the Lord Mayor stole his collar of gowld,
And sowld it away to a traitor.'"

He is manifestly wrong; but, as he very rationally observes, "no matter for that—she's a saint anyway." The "Zoist" has omitted this etymological notification! The legend however happens to come in very *apropos*, it begins

"Odille was a maid of a dignified race,
Her father, Count Otto, was lord of Alsace,
Such an air—such a grace—
Such a form—such a face,
All agreed 'twere *a fruitless endeavour to trace*
In the court, or within fifty miles of the place."

The self-same difficulty—the same "fruitless endeavour to trace" its origin attends the existence of the Baron Reichenbach's "Od Force." The "Lay of St. Odille" is droll enough; but the "Zoist" should have remembered "the Lady Odille" "was quite nervous with fear," which may account for many of the Odillic luminous phenomena seen by the baron's nervous patients. The termination of the several stanzas applied amusingly enough to this alleged "Od Force."

"Many ladies in Strasburg were beautiful; still
They were beat all to sticks by the lovely Odille.
* * * * * *
He gained the old count, who said, 'Come, Mynheer, fill;
Here's a health to yourself and the lovely Odille.'
* * * * * *
And of all whom they met, high or low, jack or jill,
Asked, 'Pray have you seen any thing late of Odille?'
* * * * * *
'Twas her voice! But 'twas *vox et preterea nil*—
Nor could any one guess what was gone with Odille.
* * * * * *
Then burst from the mountain a splendour, that quite
Eclipsed in its brilliance the finest Bude light.
'I am really ashamed of you both; my nerves thrill
At your scandalous conduct to poor dear Odille.'"

that light dwelt in God and God in light; and, although this doctrine be now repudiated, it suggested the existence of sympathies between God and man—between man and the stars that shine down upon his pathway through this sublunary sphere, which could not fail to give rise to many fervid emotions and self-elevating aspirations. Upon this theory the system of astrology, the origin of which was coeval with astronomy, was founded; the fluid which originated in the Godhead being supposed to radiate from the planets, and diffuse itself through the vital system of man, modifying according to the position and aspect of the planet which transmitted it all the functions. It is clear that medicine was at this period considered an astronomic or astrologic science; every part of the body was considered to be under the influence of one particular zodiacal constellation;* every medicinal substance imbibed its virtue from a starry influence; and our hieroglyphical prefix to our prescriptions—our recipe, ℞—remains to this day the symbol of Jupiter. This belief in astrology—the assumed influence of the stars upon the body through the medium of this subtile ether—was uppermost in the mind of Mesmer, when he began the study of animal magnetism. He had, indeed, already written and defended an inaugural dissertation "On the Influence of the Planets upon the Human Body."† The existence of this universal magnetic power had, in fact, been assumed by a host of eminent philosophers, who attempted thereby to

* Manlius gives us the following description of their powers :—

"Namque Aries capiti; Taurus cervicibus hæret
Brachia sub Geminis censentur; pectora Cancro
Te scapulæ Nemæe vocant, teque ilia Virgo :
Libra colit clunes; et Scorpius inguine regnat:
Et femur Arcitenens, genua et Capricornus amavit,
Cruraque defendit Juvenis vestigia Pisces."

Astronomicon, lib. i., 119.

We see also by Chaucer's description that astrology, in his day, formed part of the study of the physician:—

"With us there was a doctour of physike;
In all this world ne was there one like him
To speak of phisike and of surgerie;
For he was grounded in astronomie.
He kept his patients in full great dell
In houses; by his magicke naturell
Well couth he fortune the ascendant
Of his image for his patient."

"There is no infirmity, or disease," says Dr. Blagrave, "whatsoever, but in a second cause proceedeth from the influence of the afflicting planets, and what infirmity soever any planet causeth, he hath herbs by sympathy to cure it." This seems to anticipate Hahnemann's doctrine, "*Similia similibus curantur*."—Astrological Physic. The True Way to Cure all Kinds of Diseases. By Joseph Blagrave, Student in Philosophy and Physic. London: 1689.

† "Isis Revelata," vol. i., p. 151.

explain the dependence and reciprocal action of bodies in general upon each other, and the phenomena of the vital organization. "They also," adds Mr. Colquhoun, "broadly and distinctly maintained the proposition that the will or imagination of man, when energetically called into action, is capable of producing certain perceptible effects, even at a considerable distance."* This fluid theory, therefore, is of considerable antiquity. For our own part, we doubt not the existence of some nervous fluid, or aura, circulating from the nervous centres to the periphery, and its reflex action; but we cannot understand how the intervention of any magnetic or odyle fluid, however exquisitely attenuated, can blend itself with mental phenomena, or in any way account for the purely subjective operations of the mind. In the infancy of analytical science bodies were considered to be simple which the progress of experimental philosophy has proved to be compound. The earths were by Sir H. Davy's galvanic battery decomposed, and proved to consist of a metallic basis united with oxygen; light has been converted into heat, and heat into light; and we are indebted to Mr. Faraday for experimentally proving the identity between electricity and magnetism. When, therefore, we come to consider the magnetic fluid, and are informed of the discovery of a still more subtile element—the odyle force or fluid—we only advance one step further; we arrive

* The soul of the world (says Cornelius Agrippa,) is "the intelligible itself." . . . . "As the powers of our soul are communicated to the members of the body by the spirit, so also the virtue of the soul of the world is diffused through all things by this quintessence, for there is nothing formed in the whole world that hath not a spark of the virtue thereof. Yet it is more, nay, most of all, infused into those things which have received or taken in most of this spirit. Now this spirit is taken in by the rays of the stars, so far forth as things render themselves conformable to them. By this spirit, therefore, every occult property is conveyed into herbs, stones, metals, animals, through the sun, moon, planets, and through stars higher than the planets." (*Op. cit.*, b. i. c. xiv. p. 33.) We also find a chapter in this book entitled "How the passions of the mind can work out of themselves upon another's body." The following passage clearly enough anticipates all that Mesmer maintained:—"Let no man wonder that the body and soul of one may, in like manner, be affected with the mind of another, seeing the mind is far more powerful, strong, fervent, and more prevalent by its motions than vapours exhaling out of bodies; neither are there wanting medicines by which it should work, neither is another body less subjected to another's mind than to another's body. Upon this account they say that a man by his affection and habit only may act upon another." (*Ibid.*, b. i. c. lxv. p. 146.) Furthermore he observes: "Our mind doth affect divers things by faith; we must, therefore, in every work and application of things, affect vehemently, imagine, hope, and believe strongly, for that will be a great help. And it is verified among physicians *that a strong belief and an undoubted hope and love towards the physician and medicine, conduce much to health, yea more sometimes than the medicine itself. For the same that the efficacy and virtue of the medicine works, the same doth the strong imagination of the physician work, being able to change the qualities in the bodies of the sick, especially when the patient places much confidence in the physician, by that means disposing himself for the receiving the virtue of the physician and the physick.* Therefore, he that works in magic must be of a constant belief, be credulous and not at all doubt of the obtaining the effects." (Occult Philosophy, b. i. c. lxiv. p. 148.) Surely this is the very language of modern mesmerism.

only at a form of matter more subtile than any hitherto known; but we are still dealing with matter, however imponderable and refined, and we cannot conceive it convertible into thought, or affecting the independent subjectivity of the mind. The interposition, as a "*tertium quid*" between mind and matter, only increases our difficulty, by adding another link to the chain of mystery. It clears up nothing—it tends to explain nothing; the odyle light itself only contributes to render "darkness visible."

We cannot conceive why the professors of Animal Magnetism should be so enamoured of this fluid hypothesis, when the immediate and indisputable action and power of the mind upon the body so palpably may account for so many of the effects which they describe. With one accord they preach a fierce crusade against the subjective or Psychological Theory; the influence of the imagination they denounce and ridicule as a heresy long since exploded; yet, in every page they write, we constantly recognise its magic power. What but imagination, sympathy, and that fatal proclivity to imitation which so many nervous people possess, rendered the mental epidemics described by Hecker so contagious? How otherwise can we account for the miraculous cures which were wrought at St. Medard on the tomb of the Abbé de Paris? "In the case of these convulsionaries," says Mr. Braid in his answer to Mr. Colquhoun, "I would beg leave to ask how a nervous or vital force could have passed from the *ashes* of the buried saint? Mental emotion, imitation and faith, or confidence, were quite adequate to account for all which was there manifested"* The magical effects of talismans, amulets, and all manner of holy relics; the fragments of the bones of martyrs; thorns from the crown of the Saviour at his crucifixion, and pieces of wood supposed to have been part of the cross itself; how, excepting by strong faith and imagination, could these inanimate substances have effected cures? Look at the frantic zeal of the Flagellants, and the wild delirium of the victims of the dancing mania, how much they could endure without any visible signs of pain, while their bodies were swathed and their waists cruelly compressed by cloths or ropes. "While dancing," says Hecker, they "neither saw nor heard, being insensible to external impressions through the senses, but they were haunted by visions, their fancies conjuring up spirits, whose names they shrieked out. Some of them afterwards asserted that they felt as if they had been immersed in a stream of blood which obliged them to leap so high. Others, during their paroxysm, saw the heaven open, and the Saviour appear enthroned with the Virgin Mary, according

* Magic, Witchcraft, &c. *Op. cit.* By James Braid. Page 5.

as the religious notions of the age were strangely and variously reflected in their imaginations."* There can be no doubt, and the scenes which occurred almost within our time among their congregations, during the preaching of Wesley and Whitfield, sufficiently prove that excessive religious fervour will give rise to ecstatic mania.† We by no means doubt the circumstantial description which the Earl of Shrewsbury has given us of the state in which he found the Ecstatica of Caldero (Maria Mörl) and the Addolorata of Capriana (Domenica Lazzari.) Neither will we gainsay the magnetic ecstasy of the prophetess of Prevorst, whose case Dr. Kerner has so fully described.‡ We have no doubt that Ignatius Loyola, Joan of Arc, the Maid of Kent, Johanna Southcote, and many other visionaries imagining themselves inspired, were in a state of ecstatic mania, a form of insanity which develops many very extraordinary mental phenomena; they saw clearly enough the visions which their imagination, even without a sleep, conjured up; they poured forth, with the volubility of the Pythoness, their prophetic rhapsodies; but they flowed, we believe, from the fountains of a diseased mind, and did not emanate from, or evince the existence of any material fluid, ethereal, magnetic, or odylic. Similar states of mental aberration are constantly seen in lunatic asylums. Who can doubt that all descriptions of enthusiasm are contagious? Notwithstanding, therefore, all that has been written against the imaginative theory, we believe that Hecker was perfectly right in ascribing the mental epidemics, of which he has given so interesting an account, to the effects of a disordered imagination. "These plagues," he observes, (the brotherhood of the Flagellants, the dancing mania, Tarantism and Tigretier,) "crept on and found abundant food in the tone of thought which prevailed in the fourteenth and fifteenth centuries, and even in a minor degree through the sixteenth and seventeenth, causing a permanent disease of the mind, and exhibiting in those cities to whose inhabitants they were a novelty, scenes as strange as they were detestable."§ We use the word "imagination," however, in its most comprehensive sense; for, as Sir William Hamilton has philosophically remarked, "it should always be remembered, that the various mental energies are all only possible in

* Hecker. Epidemics of the Middle Ages. Sydenham Society. London :1844. Page 88.

† Life of Wesley. By Robert Southey. Vol. i., pp. 245 *et seq.*

‡ Much curious information on this subject will be found in Meric and Casaubon's Treatise on Enthusiasme. London: 1655. The cases to which he refers, he tells us, "verified the saying of Plato, that whereas the souls of ordinary men were placed in their bodies, but the bodies of holy men and philosophers were placed in their souls (p. 128)." We are told of the Prophetess of Prevorst, that "she belonged to a world of spirits. She was half spirit herself. . . . . Her body clothed her spirit like a thin veil."

§ Hecker. *Op. cit.*, pp. 88-91.

and through each other; and that our psychological analyses do not suppose any real distinction of the operations which we discriminate by different names. Thought and volition can no more be exerted apart, than the sides and angles of a square can exist separately from each other."* The same may be said of thought and imagination; it is the mind as a whole, in a state of intense concentration, which acts so powerfully on the physical organism.† Innumerable instances have been recorded shewing the influence of the imagination upon the body. The following is one of these affecting anecdotes. "Two young men intending to play a practical joke upon their sister, borrowed a skeleton from a neighbouring surgery and placed it in her bed-room. When she retired to bed they listened, expecting to hear a sudden scream; they were disappointed and retired to rest, wondering at her self-possession; but when the servant entered the room in the morning she was found playing with the skeleton in a state of complete fatuity." But imagination on the other hand may be conducive to health and happiness, and even surround us with agreeable illusions, in illustration of which Mr. Colquhoun cites a very affecting story from "Kotzebue's Journey to Paris." The sympathy between stringed musical instruments is well known even to watchmakers. "A young lady used to play on the harpsichord while her lover accompanied her on the harp. The young man died, and the harp remained in her room. After the first excess of her despair, she sank into the deepest melancholy, and some time elapsed before she could again sit down to her instrument. At last she did so, gave some touches, and hark! The harp, tuned alike, resounded in echo. The poor girl was at first seized with a secret shuddering, but soon felt a kind of soothing melancholy. She became firmly persuaded that the spirit of the lover was softly sweeping the strings of the instrument. The harpsichord from this moment constituted her only pleasure as it afforded to her mind the certainty that her lover was still hovering about her. One of those unfeeling men who want to know and clear up very thing, entered her apartment, the girl begged him to be

* Reid's Collected Writings. *Op. cit.*, p. 242.

† Hence the acute pain often caused by intense thinking. The concentrated action of the mind doubtless affecting the delicate nervous tissue of the brain. Deep study and unremitting application will produce mental derangement. So will constant reflection on any very great domestic calamity—

"That way madness lies."

In the "Fair Penitent," Rowe beautifully describes this painful dominion of thought—

"Turn not to thought, my brain; but let me find
Some unfrequented shade; there lay me down,
And let forgetful dulness steal upon me
*To soften and assuage* this pain of thinking."

quiet, for at that very moment the dear harp spoke most distinctly. Being informed of the amiable illusion which overcame her reason, he laughed; and, with a great display of learning, proved to her by experimental physics that all this was very natural. From that instant the young lady grew melancholy, drooped, and soon after died."*

But we exceed our limits. The "Letters on Animal Magnetism," by Dr. Williams Gregory, evince an unbounded belief in all the highest mysteries of animal magnetism, which he himself practices with much enthusiasm; a circumstance that calls our attention—albeit we do not wish to cavil about trifles—to Mr. Colquhoun's very unjust reflections against the medical profession. He accuses its members of "distinguishing themselves throughout by their virulent opposition to the new discovery," which he attributes to "very obvious, although not very generous or creditable, motives." He furthermore states that "our present generation of doctors and professors, however skilful in the mere technicalities of their art, and however learned in all the knowledge of a meagre, material, and narrow-minded system of philosophy, are, for the most part, utter sceptics and infidels in regard to the influence of any spiritual powers over the modifications and manifestations of the human organism."* We marvel exceedingly that Mr. Colquhoun should, under the circumstances of the case, cast such aspersions on the medical profession, when, according to his own showing, a very great number of physicians, many of whom are the most eminent men in the profession abroad and at home, have warmly espoused his cause. He appeals to works in favour of animal magnetism by Doctors Wienhoft, Treviranus, Hufeland, Gmelin, Kluge, Ennemoser, Passavant, Brandis, Zierman, &c., and others by Professors Kieser, Eschenmayer, Naas, Neesvon, Esenbeck, &c.; also to the testimony of MM. de Puysegur, Tardy de Montravel, Deleuse, Dupotet Bertrand, Roullier, Cloquet, Chardel, Rostau, Georget, Filassier, &c.; and among our English medical practitioners who have avowed their faith in animal magnetism, he refers to Doctors Elliotson, Herbert Mayo, Professor Gregory; and we could ourselves supply him with a catalogue of the names of medical men of less eminence who have become converts—besides those who are canonized in the "Zoist;" yet does Mr. Colquhoun turn his back upon these, the very authorities he himself cites as the chief pillars which support, and should induce us to enter, his temple, and accuses the whole profession of being incurably infected with scepticism and infidelity. The charge is an old one brought against us in the days of Chaucer; but it is

* Isis Revelata. Vol. i. p. 169.
† History. Vol. i., preface, p. 9,—p. 88.

utterly untrue, and we emphatically repudiate it.* The most eminent men of the present generation—ay, and of the past, too, else why does Mr. Colquhoun fall back on the authorities of Hippocrates, Galen, Aratæus, Paracelsus, and Van Helmont?—have always been too much addicted to theorise and speculate upon our spiritual nature. The spirit which suggested the solution of abstract questions among the scholastic philosophers has ever been busy among us; and even young men in the dissecting room become so easily led astray into these fascinating speculations, that our professors are continually called upon to admonish them and bring them back to the strict principle of induction propounded by the Baconian philosophy. All the most eminent physicians of the present age have avowed their adherence to the faith of Christianity, and recognised to the fullest extent the spiritual ascendancy of man. The late Sir Henry Halford, Mason Good, Abercrombie, Charles Bell, Monro, Cooper; and among our living physicians, Chambers, Holland, and hundreds of others who hold a distinguished position in the profession—in fact, all the members of the medical profession, are thus far spiritualists in the discharge of their professional duties: they so clearly recognise the distinction and reciprocal action between mind and matter, that not one, we feel assured, would ever prescribe for any patient without duly considering the moral and mental causes which may aggravate, or complicate, the symptoms of the disease. But we are afraid the professors of animal magnetism are an irritable race; perhaps the propounders of all new doctrines become afflicted with what Southey calls "a mimosa sensibility;" they cannot endure the idea of being contradicted, even in their theories, when they have agreed upon their facts; they will not allow any counter opinions even to be thrown into the scale of evidence, and, like Sir Anthony Absolute, abuse every body who differs from them, while they imagine themselves to be in a very cool and equable state of temper. In the very last number of the "Zoist" (April, 1852, No. xxxvii.) we find Dr. Elliotson, in some remarks annexed to a very interesting paper by Professor Gregory, on the "Theory of Imagination," apply the following ungentle language to Sir David Brewster, Professor Forbes, Professor Bennett, Professor Goodson, and Dr. Simpson, whose names, to prevent any mistake, he thus sig-

* Chaucer taxed the faculty with irreligion, in his description of the Physician, given above,—

"Of his diet, measureable was he,
For it was of no superfluitie,
But of great nourishing and digestible.
*His study was but little on the bible.*

The late Dr. Millingen, in his "Curiosities of Medical Experience," cites these lines, and refers to the old adage "*Ubi tres Medici-duo Athei.*" Assuredly this would be held to be a calumny by the physicians of the present age.

nally *taboos:*—"From them no benefit has ever accrued to mesmerism, nor is likely ever to accrue. They are doggedly insensible to its splendid facts; their hearts are hardened, and their intellect thereby stupified" (page 33). Hard words, gentlemen! hard words! and the sooner Dr. Elliotson contrives to subdue these refractory philosophers by mesmerizing them *in distans* the better. Then, again, we find Mr. Colquhoun and Mr. Braid involved in a pleasant little schism—engaged, as the clown in the forest describes to Audrey, upon a cause "seven times removed"—the "counter-check quarrelsome." Unluckily, Mr. Colquhoun somewhat inadvertently accused Mr. Braid of being a materialist, and thereupon Mr. Braid retorts upon Mr. Colquhoun that he has not an orthodox faith in the actual personality and incarnate existence of the devil; wherefore he, in round terms, charges Mr. Colquhoun with not being a true Christian. "*Tantæne cœlestibus iræ?*" All this we conceive to be very absurd. The world at large cares little for the squabbles of men of science; they may sit within the shadow of their own academic groves and blow any soap-bubbles they please, but when they come forward to propound the principles of any theories which are, and always have been, so deeply interesting to all reflecting men, they should not so far detract from what we consider should ever be the self-possessed and true dignity of philosophy. When men lose their temper, they betray their weakness. All we want is to be assured of the existence of *facts* upon which we can build something like a satisfactory and enduring faith.

We regret we have not space to pursue this subject—which is sufficiently enticing—further. We conceive that the scientific world is indebted to Mr. Colquhoun for his attempting to pioneer us through these perplexing mysteries; even those who dissent from him, and are opposed to his views, will not rise from the perusal of the "Isis Revelata," and "The History of Magic, Witchcraft, and Animal Magnetism," without having derived a vast amount of information. But, we repeat, the whole subject is involved in much obscurity and difficulty; and, as we commenced by citing a passage from the Rev. Sidney Smith's "Elementary Sketches of Moral Philosophy," so shall we conclude by subjoining another extract, which needs no comment, from these very spirited and charming lectures. In alluding to the department of metaphysics, which comprehends psychological science, he observes: "If there be a real foundation for this science—if observation can do anything, and has not done all—there is room for hope and reason for exertion. The extravagances (alluding to the Berkleian theory, the non-existence of matter; but the remark applies particularly to animal magnetism)—the extravagances by which it has been disfigured ought to warn us of the difficulty, without leading us to despair. To say there is no path, because we have often got into the wrong path, puts an end to all other knowledge as well as this. The truth is, it fares worse with this science than with many others, because its errors and extravagances are comprehended by so many."*

---

### Art. IV.—ON MENTAL PHYSIOLOGY.†

* Elementary Sketches. *Op. cit.* Lecture i. p. 5.

